# Whole-genome resequencing reveals genomic characteristics and candidate genomic regions potentially associated with local adaptation of Leiwuqi yak (*Bos grunniens*)

**DOI:** 10.3389/fvets.2026.1902267

**Published:** 2026-07-29

**Authors:** Chenbo Shi, Lin Fu, Tengxiang Wang, Jia Zhou, Yu Zeng, Li Zhang, Xuefeng Guan, Gaofu Wang, Rui Hu, Miaoyu Zhou, Jianqing Zhao, Xianwen Dong

**Affiliations:** 1 Chongqing Academy of Animal Sciences, Chongqing, China; 2 Animal Nutrition Institute, Sichuan Agricultural University, Chengdu, China; 3 College of Animal Science, Xinjiang Agricultural University, Urumqi, China

**Keywords:** genetic diversity, Leiwuqi yak, population structure, selective sweep, whole-genome resequencing

## Abstract

Leiwuqi yak is an important indigenous yak genetic resource distributed in eastern Tibet, but its genomic characteristics and adaptive evolutionary features remain poorly understood. In this study, whole-genome resequencing was performed on 110 Leiwuqi yaks, and these data were integrated with publicly available genomic data from other domestic and wild yak populations to investigate the genetic diversity, population structure, and candidate genomic regions potentially associated with local adaptation of Leiwuqi yak. After quality control and variant filtering, a total of 19,966,141 high-quality SNPs were identified across all samples. Most SNPs were located in intronic and intergenic regions, with a transition/transversion ratio of 2.47. Although sequencing depth differed between newly sequenced (3.87×) and public (9.04×) data, all samples were processed through a unified pipeline with stringent filtering criteria. Genetic diversity analyses showed that Leiwuqi yak retained relatively abundant nucleotide diversity, whereas runs of homozygosity and genomic inbreeding coefficient analyses suggested possible effects of local isolation or recent inbreeding. Population structure analyses based on principal component analysis and ADMIXTURE revealed that Chinese domestic yak populations shared a broadly similar genetic background with wild yak, whereas Indian yak exhibited clear genetic differentiation. Although Leiwuqi yak did not form a completely independent genetic cluster at the genome-wide level, selective sweep analysis identified localized genomic differentiation in this population. A total of 466 protein-coding genes were detected within candidate selected regions. Functional enrichment analyses showed that these genes were mainly associated with the Wnt signaling pathway, NF-kappa B signaling pathway, pathways in cancer, light absorption, and receptor-mediated endocytosis. These findings suggest that developmental regulation, immune and stress responses, environmental perception, and cellular homeostasis may contribute to the adaptive differentiation of Leiwuqi yak. Overall, this study provides new genomic evidence for understanding the genetic uniqueness and adaptive evolution of Leiwuqi yak and offers a scientific basis for its conservation and sustainable utilization.

## Introduction

Yaks (*Bos grunniens*) represent a distinctive species predominantly inhabiting the Qinghai-Tibet Plateau and its adjacent regions, such as the Himalayan and Kunlun Mountains. These habitats are typified by high altitudes, low temperatures, hypoxic conditions, and intense ultraviolet radiation (1–4). Historically, yaks have been integral to the livelihoods of herders in alpine pastoral areas, which are characterized by sparse vegetation and thin air, by providing a reliable source of meat, milk, and fur. Additionally, they fulfill essential roles in labor and transportation, thus playing a pivotal role in past oral cultural systems (5–7). The domestication of yaks can be traced back approximately 7,000 years (8). Over the millennia, domestic yaks have been subject to extensive natural selection and artificial breeding (9), resulting in the emergence of numerous local breeds with distinct morphological characteristics and productivities (5, 10–12). The extensive distribution of yaks throughout the Qinghai-Tibet Plateau and its surrounding regions is of considerable significance to the local ecosystem and global biodiversity conservation efforts. Importantly, yaks demonstrate variations in genetic diversity and population structure across distinct geographical areas. These findings offer a crucial scientific foundation for the conservation and sustainable management of yak (8, 13–15) resources.

Research on the yak genome provides valuable insights into species evolution and adaptation, while also playing a critical role in harmonizing the development of animal husbandry with human health considerations. Recent advancements in whole-genome resequencing technology and the assembly of high-quality genomes have enabled a more comprehensive mapping of the genetic diversity of yaks (16), positioning genome-wide studies based on whole-genome resequencing as a prominent area of research. Through whole-genome resequencing of six yak breeds from Sichuan, Yunnan, and Tibet, researchers have identified significant variations in genomic diversity and population structure among these breeds. Furthermore, positive selection signals were detected in candidate genes associated with immunity, hypoxia adaptation, and pigmentation (17). Analysis of genomic data from various yak populations has also led to the identification of candidate genes related to lipogenesis, energy metabolism, and reproduction, which are likely to form the genetic foundation for yaks’ adaptation to harsh environments (18). Through the application of high-density SNP genotyping and whole-genome sequencing technologies, it has been determined that the introgressed segments originating from bovine ancestors within the Mongolian yak genome are enriched with genes linked to the development and function of the nervous system. Notably, these segments exhibit a significant concentration in pathways related to glutamate metabolism and neurotransmission (19). These findings indicate that whole-genome resequencing is a powerful approach for revealing population differentiation, adaptive loci, and the genetic mechanisms underlying adaptation in yaks.

Leiwuqi yak is a local yak genetic resource mainly distributed in Leiwuqi County, Changdu City, Tibet, located in the eastern part of the Qinghai–Tibet Plateau. This region is characterized by a complex plateau environment, including high altitude, large diurnal temperature variation, intense solar radiation, and seasonal forage shortage. As an important livestock resource for local herders, Leiwuqi yak plays a significant role in meat and milk production, transportation, and the maintenance of traditional pastoral livelihoods. However, compared with several well-studied yak breeds, the genomic background, genetic diversity, population structure, and adaptive evolutionary characteristics of Leiwuqi yak remain poorly documented.

In this study, we performed whole-genome resequencing to investigate the genomic characteristics of Leiwuqi yak and to evaluate its genetic relationship with other domestic yak populations. We systematically analyzed genome-wide single nucleotide polymorphisms, genetic diversity, population structure, and linkage disequilibrium patterns. In addition, to identify potential population-specific candidate genomic regions potentially associated with local adaptation, we integrated nucleotide diversity ratio and population differentiation coefficient analyses to detect candidate selected regions in Leiwuqi yak. Functional annotation and enrichment analyses were then conducted for genes located within these regions to explore their potential biological roles. This study provides new genomic evidence for understanding the genetic uniqueness and adaptive evolution of Leiwuqi yak, and offers a scientific basis for the conservation, evaluation, and sustainable utilization of this valuable yak genetic resource.

## Materials and methods

### Sample collection and sequencing

Blood samples from 110 Leiwuqi yaks (LWY, *n* = 110) were collected in Leiwuqi County, Changdu City, Tibet Autonomous Region, China, and preserved in liquid nitrogen. Genomic DNA was extracted using the standard phenol-chloroform method. DNA quality and concentration were assessed before library construction. Qualified DNA samples were used to construct paired-end libraries with an average insert size of 350 bp and an average read length of 150 bp (Illumina PE150), followed by whole-genome sequencing by Beijing Allwegene Gene Technology Co., Ltd. In addition, 62 publicly available yak genomes representing five populations were obtained from public databases, including Tianzhu yak (TZY, *n* = 7), Datong yak (DTY, *n* = 8), Hongyuan yak (HYY, *n* = 5), Indian yak (IDY, *n* = 30), and wild yak (WY, *n* = 12). Together with the 110 newly sequenced Leiwuqi yaks, a total of 172 individuals were included in this study.

All procedures involving animals were conducted in accordance with the guidelines for the care and use of experimental animals and were approved by the Animal Ethics Committee of Chongqing Academy of Animal Sciences.

### Read mapping, variant calling, and SNP annotation

In this study, paired-end sequencing data were processed using the fastp tool (v 0.20) (20) to obtain high-quality reads. The clean reads were then aligned to the yak reference genome LU_Bosgru_v3.0 (GCA_005887515.1, https://www.ensembl.org/index.html) utilizing the BWA tool (v 0.7.17-r1188) (21), and BAM files were subsequently generated with Samtools (v 1.21) (22).

The SortSam module of the Picard tool (v 2.20.4) was employed to sort the BAM files (23), and the MarkDuplicates module was used to eliminate potential duplicate reads. Indexing of the sorted BAM files was performed with Samtools, and alignment rate analysis was conducted using the flagstat module of Samtools. Additionally, the bamqc module of QualiMap (v2.2.2-dev) was used to evaluate sequencing depth and coverage (24).

Following read alignment and post-processing, variant calling was performed using the HaplotypeCaller module of the Genome Analysis Toolkit (GATK, v 4.3.0.0) in GVCF mode for each individual (25). Individual GVCF files were then jointly genotyped using the GenomicsDBImport and GenotypeGVCFs modules to generate a raw population-level VCF file. SNPs and InDels were separated using SelectVariants and filtered independently.

For SNP filtering, hard-filtering was conducted using the VariantFiltration module of GATK with the following criteria: QD < 2.0, FS > 60.0, MQ < 40.0, MQRankSum < −12.5, and ReadPosRankSum < −8.0. For InDel filtering, variants were removed if they met any of the following criteria: QD < 2.0, FS > 200.0, or ReadPosRankSum < −20.0. Variants located in dense clusters were further filtered using vcfutils.pl. with the parameters “-w 5 -W 10,” which removes variants located within 5 bp of an InDel or within clustered variant regions, thereby reducing potential false-positive calls caused by local misalignment. Subsequently, additional population-level filtering was performed using VCFtools (v 0.1.16) (26). Only biallelic SNPs located on autosomes were retained. SNPs were further filtered using VCFtools with the following criteria: minor allele frequency (--maf) ≥ 0.05, minimum quality score (--minQ) ≥ 30, depth (--minDP) ≥ 2 and (--maxDP) ≤ 1,000, and a maximum missing rate of 30% (--max-missing 0.7). After these filtering steps, high-quality SNPs were retained for subsequent population genomic analyses. Functional annotation of the filtered SNPs was performed using SnpEff software based on the gene annotation file of the yak reference genome LU_Bosgru_v3.0. SNPs were classified according to their genomic locations and predicted functional effects, including intergenic, intronic, exonic, upstream, downstream, untranslated region, synonymous, missense, stop-gained, stop-lost, and splice-site variants.

### Genetic diversity analysis

This study subsequently examined the genomic variation characteristics of various yak breeds, including LWY, TZY, DTY, HYY, IDY and WY. The analysis focused on nucleotide diversity (*π*), linkage disequilibrium (LD) decay, and runs of homozygosity (ROH). LD decay was estimated using the filtered high-quality SNP dataset. The average *r*^2^ between SNP pairs was calculated as a function of physical distance using PLINK software with the parameters: --ld-window 100 --ld-window-kb 100 --ld-window-r2 0. LD decay curves were generated using the ggplot2 package in R software (v 4.2.0) (27) and LD decay curves were generated using the ggplot2 package in R software (v 4.2.0). To calculate *π* values from the original VCF files, VCFtools (v 0.1.16) was employed with a sliding window approach, using a window size of 100 kb and a step size of 10 kb (26). *π* values were calculated for each yak breed/population individually, and the results were visualized through the creation of Manhattan plots and smoothed line plots using R software (v 4.2.0). The detection of ROH was performed using PLINK software (v 1.9) with the following parameter settings: sliding windows consisted of 50 SNPs, allowing up to 1 heterozygous call and 5 missing calls per window; a minimum of 30 consecutive homozygous SNPs was required to define a ROH; the minimum length of a ROH was set to 300 kb; the minimum density threshold was set to 1 SNP per 100 kb; and the maximum allowed gap between consecutive SNPs within a ROH was 1,000 kb. In addition, analyses were run with chromosome set to 29 and allowing extra chromosomes, while no minor allele frequency (MAF) filter was applied. The number and total length of ROHs were calculated for each individual, and ROHs were subsequently classified into four length categories: 0.3–0.5 Mb, 0.5–1 Mb, 1–2 Mb, and >2 Mb, corresponding to ancient, historical, and recent inbreeding events, respectively. Genomic inbreeding coefficient *F*_HOM_ for each individual was calculated from genome-wide SNP data by comparing observed heterozygosity with expected heterozygosity, to assess individual genomic inbreeding status. Genome-wide *π* values was calculated using a sliding-window approach. Genomic regions with *π* values exceeding the 99th percentile of the genome-wide distribution were defined as high-*π* regions and subjected to gene annotation. All graphical representations were generated using R software.

### Population structure analysis

Principal component analysis (PCA) was performed using PLINK v1.9 (28) and visualized with the ggplot2 package in R software. First, LD pruning was conducted in PLINK to generate a dataset of low-LD loci. Subsequently, ADMIXTURE v1.3.0 (29) was used to assess population structure and determine the optimal *K* value, and the results were visualized using R software.

### Detection of selective sweeps

Using the filtered high-quality SNP dataset, two complementary statistics, *F*_ST_ and the nucleotide diversity ratio *θπ*, were used to identify candidate selective sweep regions in LWY. *F*_ST_ values between LWY and other domestic yak populations (ODY, including DTY, HYY, IDY, and TZY) were calculated using VCFtools (v0.1.16) with a sliding window size of 100 kb and a step size of 10 kb (26). *θπ* values were calculated separately for LWY and ODY using the same sliding-window approach, and the log2-transformed *θπ* ratio was calculated as log2_(*θπ*_LWY/*θπ*_ODY)_. Windows in the highest 5% of Z-transformed *F*_ST_ values and the lowest 5% of log2_(*θπ*_LWY/*θπ*_ODY)_ values were considered candidate selective sweep regions. Genes overlapping with these candidate windows were annotated as candidate genes.

### Functional enrichment analysis

OmicShare tool^1^ was used to perform and visualize KEGG (Kyoto Encyclopedia of Genes and Genomes) and GO (Gene Ontology) enrichment analyses. Pathways or terms with a *p*-value <0.05 were considered significantly enriched.

## Results

### Sequencing and variation calling

In this study, a comparative analysis was performed on whole-genome resequencing data from 172 yaks. This dataset comprises newly sequenced genomes from 110 LWY yaks, alongside genome data from 62 yaks (including HYY, TZY, DTY, IDY, and WY) obtained from public databases. For the newly sequenced LWY genomes, the average alignment rate was 97.41%, with an average sequencing depth of 3.87× and an average GC content of 43.83%. Post-filtering for low-quality sequences, the mean number of clean reads per sample was 73,460,303 reads. In contrast, the downloaded yak genomes exhibited an average alignment rate of 97.60%, an average sequencing depth of 9.04× and an average GC content of 42.81%. After the removal of low-quality sequences, the average number of clean reads per sample was 199,775,348 reads. Across all samples, the overall average alignment rate was 97.47%, with an average sequencing depth of 5.73× and an average GC content of 43.46%. Following quality filtering, the average number of clean reads per sample was 118,992,469 reads (Supplementary Table S1).

**Figure 1:**
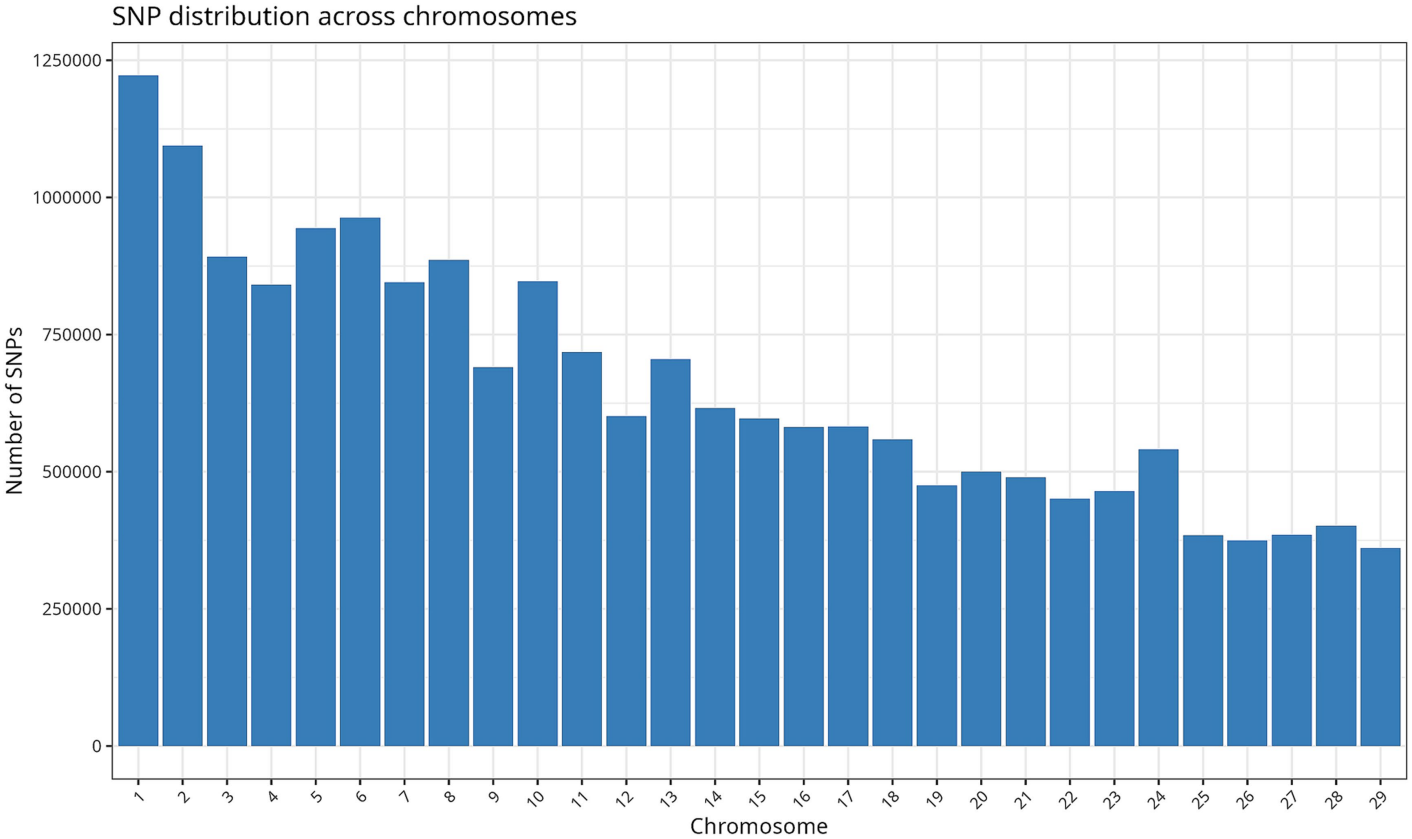
Distribution of SNP counts across 29 autosomal chromosomes in the yak genome. The *x*-axis represents chromosomes 1–29, and the *y*-axis shows the number of high-quality SNPs identified on each chromosome.

A total of 19,966,141 high-quality SNPs were identified. SNP distribution on individual chromosomes was statistically investigated (Figure 1). The majority were located in intronic (51.19%) and intergenic regions (38.51%), while exonic SNPs accounted for only 0.95% (percentages calculated relative to the total number of high-quality SNPs). Within exonic regions, we detected 173,073 synonymous and 112,332 missense mutations. The Ts/Tv ratio was 2.47, indicating reliable data quality. These results provide a comprehensive variant resource for subsequent genomic analyses (Table 1).

**Table 1:** Summary of SNP annotation and functional classification in the population.

Category	Count	Percentage (%)
Variant location		
Exonic	323,452	0.95
– Synonymous	173,073	0.51
– Missense	112,332	0.33
– Stop-gained	1,736	0.01
– Stop-lost	238	0
– Start-lost	290	0
Intronic	17,514,044	51.19
Intergenic	13,176,866	38.51
Upstream (≤2 kb)	1,467,135	4.29
Downstream (≤2 kb)	1,504,519	4.4
3′ UTR	71,647	0.21
5′ UTR	28,443	0.08
Splice site region	28,811	0.08
Splice acceptor	374	0
Splice donor	501	0
Non-coding transcript exon	39,853	0.12
Non-coding transcript	100,534	0.29
Transitions/Transvs		
Transitions (Ts)	14,219,511	–
Transvs (Tv)	5,746,630	–
Ts/Tv ratio	2.47	–

Counts in intronic and intergenic categories are transcript-level annotation records from SnpEff and may exceed the number of unique SNPs. All percentages are based on total SNPs, except for missense, synonymous, and stop-gained variants (indented under Exonic), which are based on exonic SNPs only. For multiple transcripts, only the most severe consequence per variant was retained.

### Genetic diversity analysis

**Figure 2:**
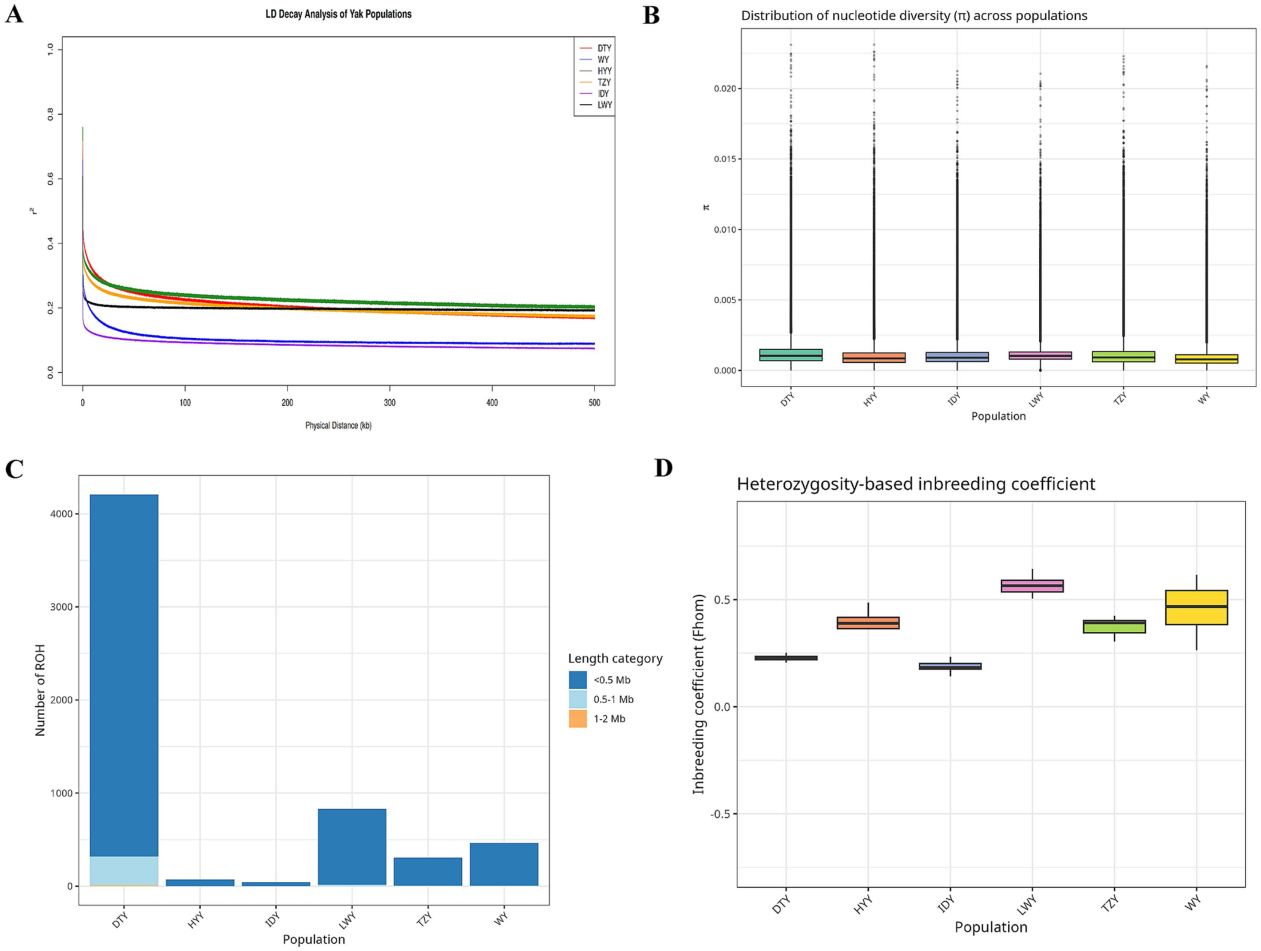
Genetic diversity among 172 samples from 6 yak populations. **(A)** Genome-wide LD decay curves for each population. The *x*-axis represents physical distance (kb) and the *y*-axis shows the mean *r*^2^ between SNP pairs. **(B)** Nucleotide diversity (*π*) distribution across populations. The *x*-axis shows the six populations and the *y*-axis shows *π* values calculated. **(C)** Total number of ROH per individual across populations. The *x*-axis shows the six populations and the *y*-axis shows the number of ROH per individual. **(D)** Genomic inbreeding coefficient (*F*_HOM_) per individual. The *x*-axis shows the six populations and the *y*-axis shows *F*_HOM_ values.

The rapid LD decay observed in IDY and WY may reflect relatively high genomic diversity, weaker recent artificial selection, or distinct demographic histories (Figure 2A). Nucleotide diversity analysis (Figure 2B) indicated relatively high genetic diversity in DTY and LWY. ROH, an important metric for evaluating relatedness and inbreeding history, reflect recent genomic evolution and demographic history. Analysis of short ROH fragments showed that ancient inbreeding was more common in DTY and LWY. Moreover, DTY exhibited a higher overall ROH content, implying a longer history of domestication and selective breeding (Figure 2C). *F*_HOM_ results revealed that LWY had the highest inbreeding coefficient, while IDY had the lowest (Figure 2D).

**Figure 3:**
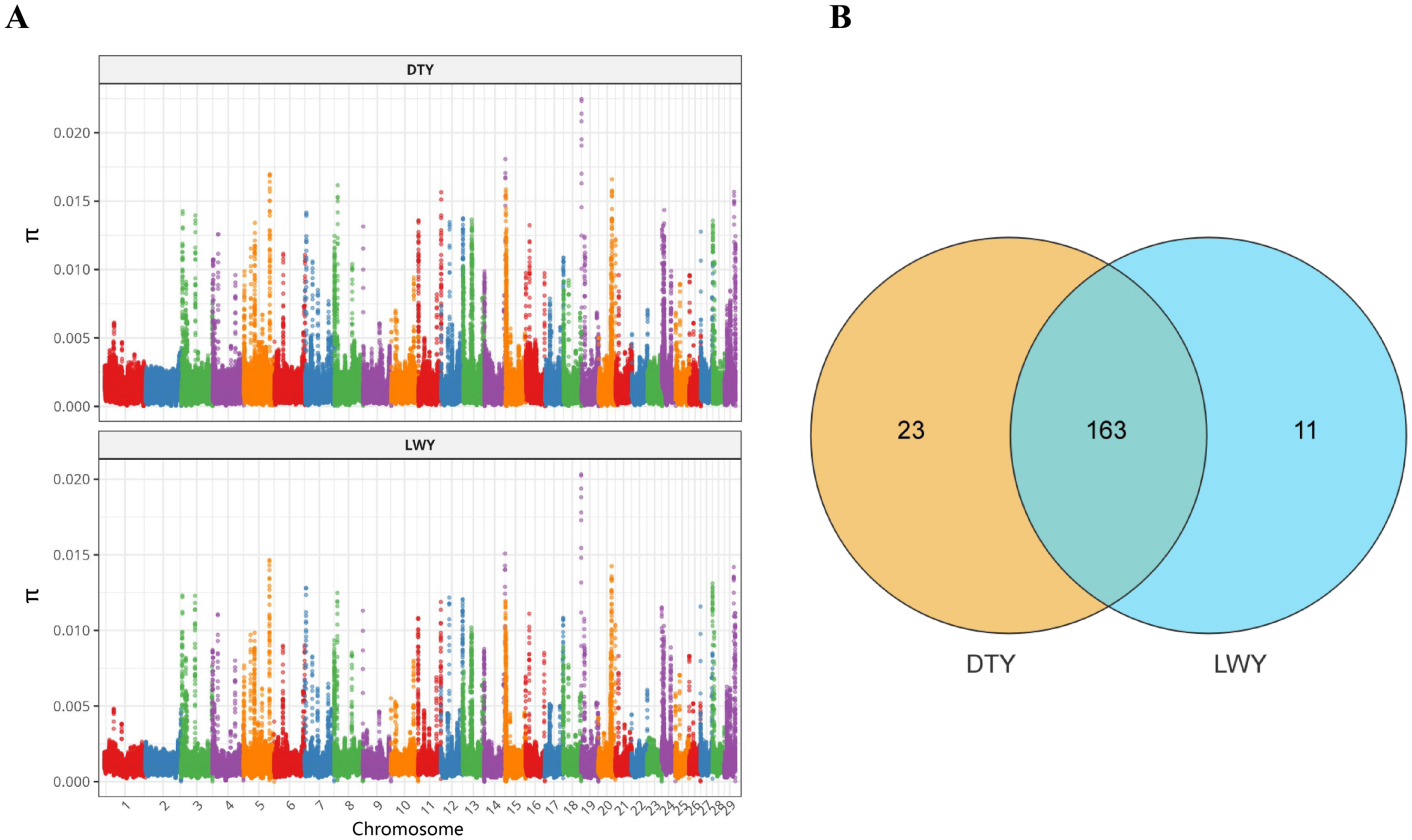
Genomic regions with elevated nucleotide diversity in DTY and LWY. **(A)** Manhattan plot of nucleotide diversity (*π*) for DTY. **(B)** Venn diagram of annotated genes overlapping with top 1% *π* windows in DTY and LWY. Numbers indicate gene counts: DTY-specific (23), shared (163), and LWY-specific (11).

**Figure 4:**
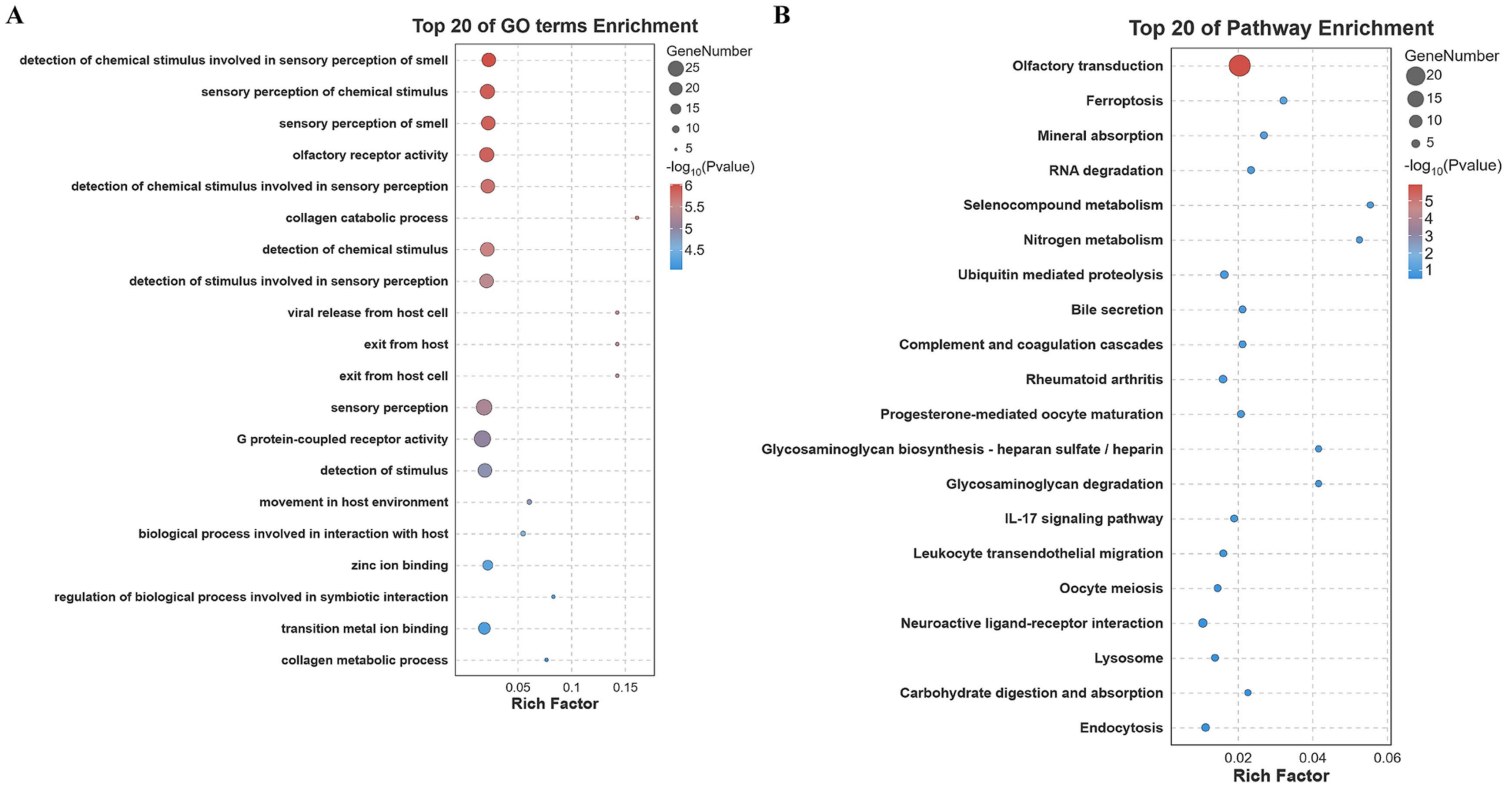
Functional enrichment analysis of genes shared between high-*π* regions of DTY and LWY. **(A)** GO enrichment analysis of shared high-*π* genes. The *x*-axis represents the rich factor, and the *y*-axis shows the top 20 enriched GO terms. **(B)** KEGG pathway enrichment analysis of the same gene set. The *x*-axis shows the rich factor, the *y*-axis lists the top 20 enriched pathways.

Manhattan plot analysis identified 2,661 windows (top 1% of the genome-wide distribution) with elevated nucleotide diversity in DTY, which overlapped with 186 annotated genes (Figure 3A). Similarly, 2,661 high-*π* windows were identified in LWY, overlapping with 172 genes (Figure 3A). A total of 163 genes were shared between the two breeds, including MMP1, LOXL2, and PGR (Figure 3B). GO enrichment analysis of the shared genes between DTY and LWY revealed significant enrichment of terms linked to olfactory perception and chemical stimulus detection, including sensory perception of smell, olfactory receptor activity, and chemical stimulus detection (*p* < 0.05) (Figure 4A). In contrast, only the Olfactory transduction KEGG pathway was significantly enriched (*p* < 0.05) (Figure 4B).

### Population genetic structure

**Figure 5:**
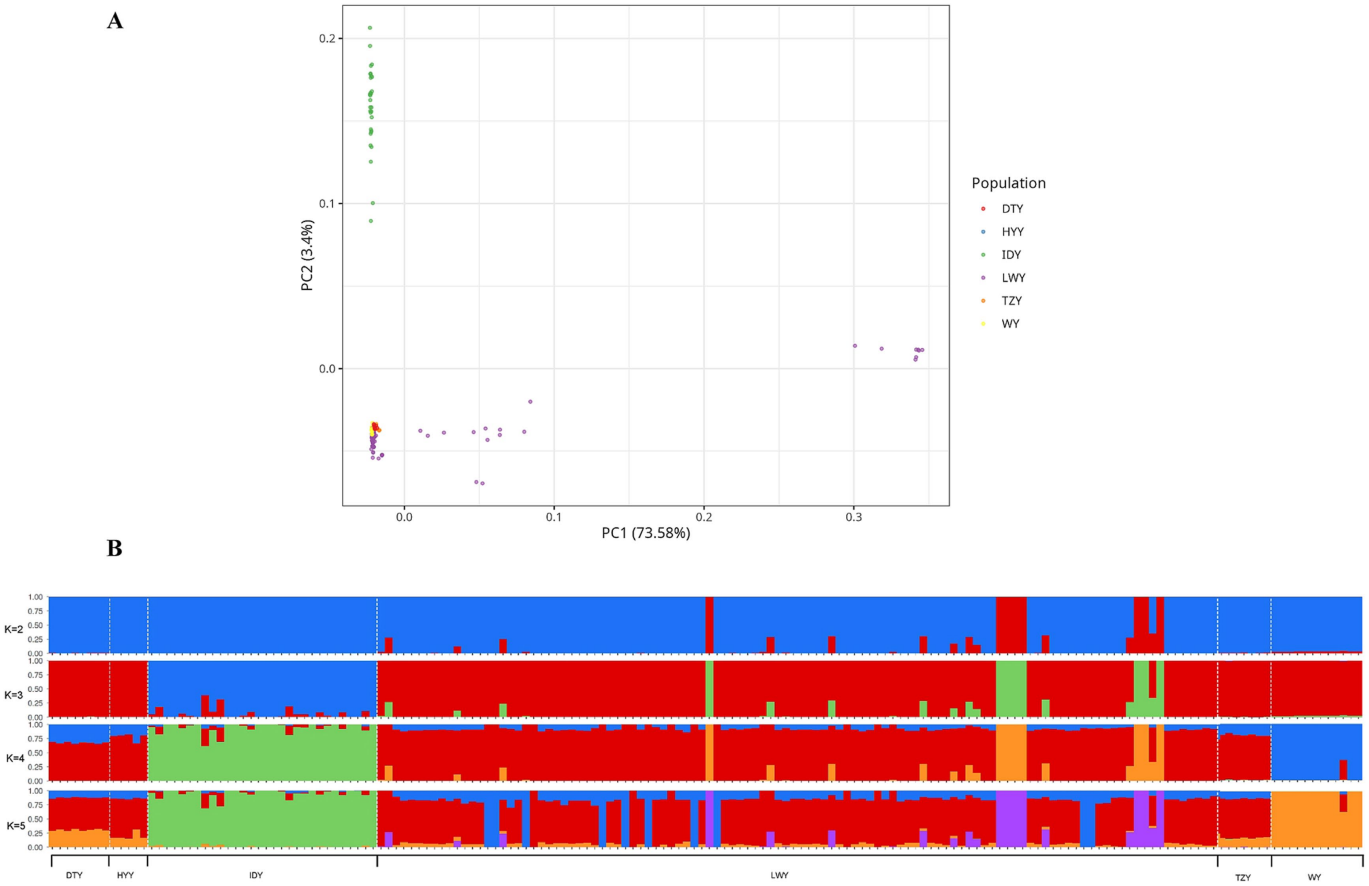
Population genetic structure of six yak populations. **(A)** PCA of individuals based on genome-wide SNPs. The *x*-axis represents PC1 (73.58% of total variance) and the *y*-axis represents PC2 (3.4% of total variance). **(B)** ADMIXTURE analysis for *K* values from 2 to 5.

To investigate the genetic structure of the six yak breed populations, population genetic analyses were performed using SNP markers derived from whole-genome resequencing. Principal component analysis (PCA) showed that the first and second principal components explained 73.58 and 3.4% of the total variance, respectively (Figure 5A). Chinese domestic yaks (LWY, TZY, HYY, and DTY) largely overlapped with wild yaks and could not be clearly distinguished. In contrast, Indian yaks (IDY) formed a separate cluster and exhibited clear genetic differentiation from all Chinese yak germplasm resources. No distinct genetic separation was detected among the native Qinghai–Tibet Plateau domestic yak breeds or between them and wild yaks.

Furthermore, admixture analysis was performed with the number of ancestral subpopulations (*K*) set from 2 to 10. Cross-validation was used to determine the optimal number of clusters, which was identified as *K* = 3 based on the lowest cross-validation error rate (CV errors for *K* = 2 to 10 were 0.57338, 0.55434, 0.56437, 0.57892, 0.59890, 0.62444, 0.64359, 0.66336 and 0.67922, respectively). Each yak population possessed distinct ancestral genetic components, and clear genetic differentiation was observed among populations. At all tested *K* values, a clear genetic boundary was consistently observed between Chinese indigenous yaks and Indian yaks, indicating substantial differences in genetic composition among the groups. By contrast, the overall genetic background of Chinese indigenous yak germplasm resources was relatively homogeneous (Figure 5B).

### Selective sweep test

**Figure 6:**
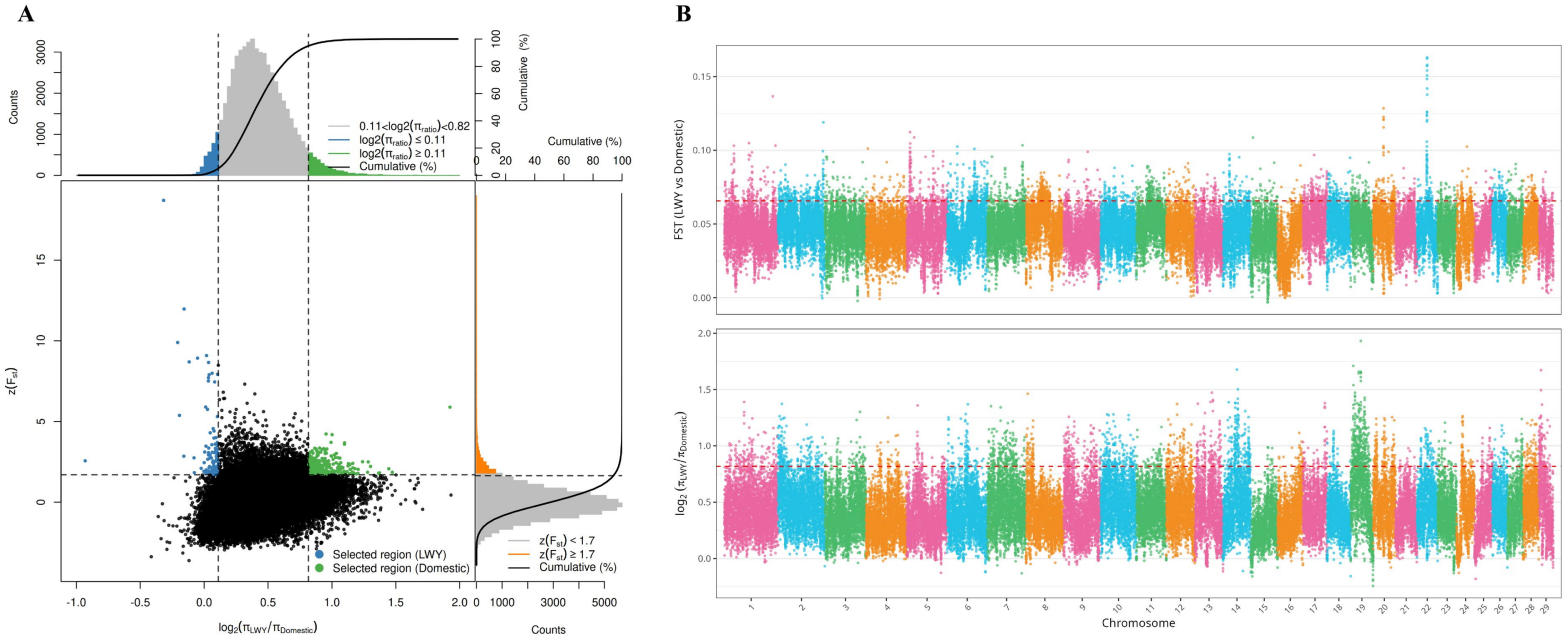
Identification of selective sweep regions in LWY relative to other ODY. **(A)** Distribution of *Z*(FST) and log_2_(*θπ*(LWY/ODY)) between LWY and ODY. **(B)** Manhattan plot of *F*_ST_ (LWY/DY) and log_2_(*π*LWY/*π*ODY).

**Figure 7:**
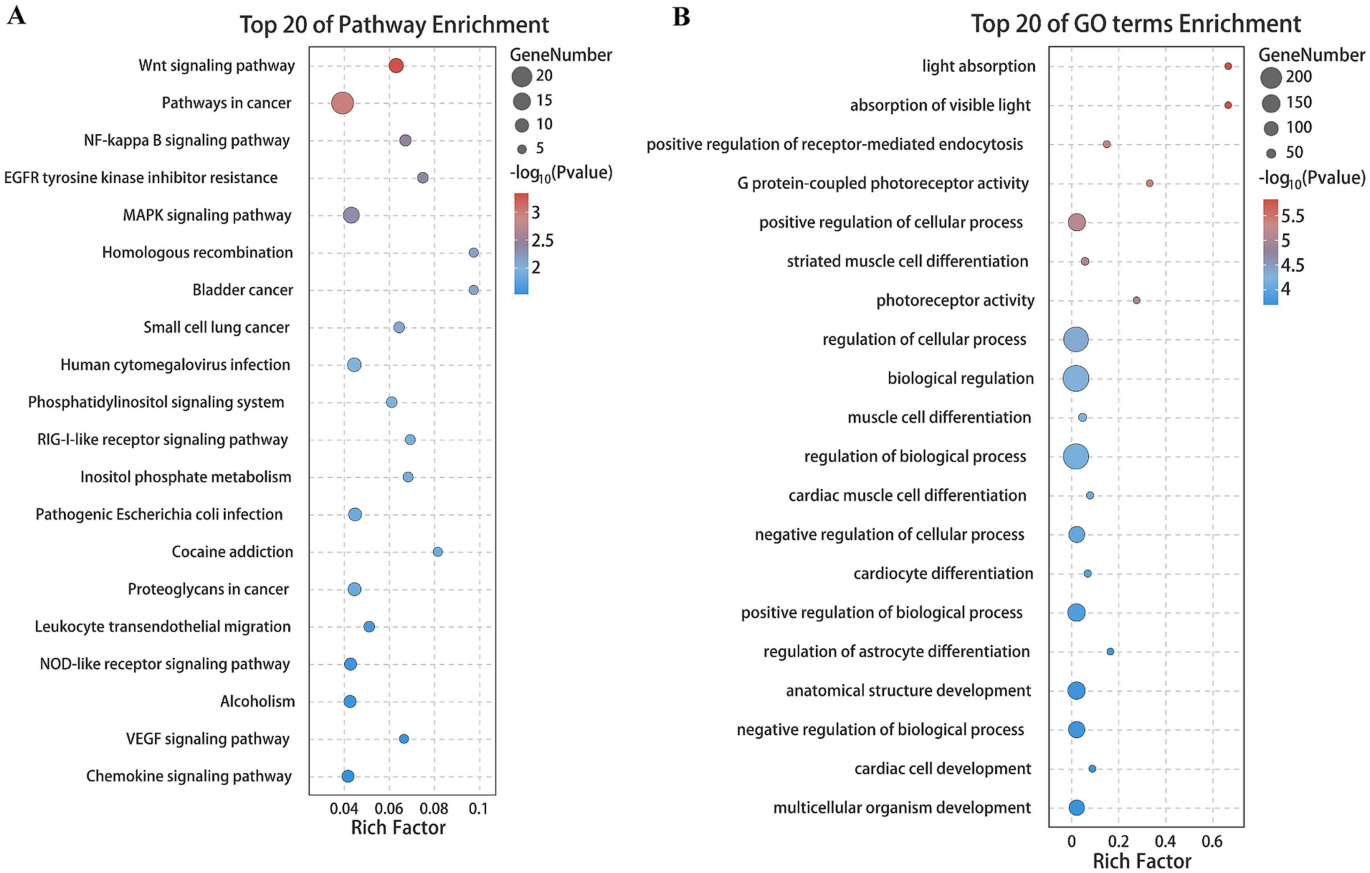
Functional enrichment analysis of genes located in candidate selective sweep regions in LWY. **(A)** KEGG pathway enrichment analysis. The *x*-axis represents the rich factor and the *y*-axis lists the top 20 enriched KEGG pathways. **(B)** GO enrichment analysis of the same gene set. The *x*-axis shows the rich factor, the *y*-axis lists the top 20 enriched GO terms.

To identify genomic regions potentially under selection in LWY relative to other domestic yak populations (ODY), we performed a comparative population genomic analysis based on *F*_ST_ and the *θπ* ratio. Candidate selective sweep regions were defined using empirical thresholds corresponding to the highest 5% of *Z*(*F*_ST_) and the lowest 5% of log2_(*θπ*_LWY/*θπ*_ODY)_. Windows with log2_(*θπ*_LWY/*θπ*_ODY)_ ≤ 0.11 and *Z*(*F*_ST_) ≥ 1.7 were retained as candidate regions under positive selection in LWY (Figures 6A, B). A total of 466 protein-coding genes were annotated within these candidate regions. KEGG enrichment analysis showed that these genes were significantly enriched in the Wnt signaling pathway, pathways in cancer, and the NF-kappa B signaling pathway (Figure 7A). GO enrichment analysis showed significant enrichment of terms including light absorption, absorption of visible light, and positive regulation of receptor-mediated endocytosis (Figure 7B).

## Discussion

Yaks have undergone long-term natural and artificial selection in the Qinghai–Tibet Plateau and adjacent high-altitude regions, resulting in abundant local genetic resources with distinct ecological adaptability and production traits (30, 31). Leiwuqi yak is an important indigenous yak genetic resource distributed in eastern Tibet. Its main producing area is located in the transitional zone between the Nyainqentanglha Mountains and the alpine canyon of eastern Tibet, with an average altitude of 4,500 m. It is a vital component of domestic yak breeds. However, its genome-wide genetic characteristics remain poorly elucidated to date. In this study, we generated whole-genome resequencing data for 110 Leiwuqi yaks and integrated them with publicly available genomic data from other domestic and wild yak populations. A total of 19.97 million high-quality SNPs were identified, providing a valuable genomic resource for investigating the genetic diversity, population structure, and potential adaptive mechanisms of Leiwuqi yak.

In our study, among the 19.97 million high-quality SNPs identified from 172 yak genomes, most were located in intronic and intergenic regions, while exonic SNPs accounted for only a small proportion. The distribution of SNPs observed in this study was generally consistent with patterns reported in previous yak and cattle genome resequencing studies (32). This is expected because non-coding regions occupy a large fraction of mammalian genomes and are usually under weaker functional constraint than coding regions. By contrast, exonic variants, particularly nonsynonymous SNVs, stop-gain, stop-loss, and splice-site variants, are more likely to affect gene function and may therefore be subject to stronger purifying selection (33, 34). The transition/transv ratio was 2.47, suggesting that the SNP dataset was not dominated by random sequencing errors and supporting the reliability of the variant calling and filtering procedures (35, 36). These results suggest that the final SNP set was reliable for downstream population genomic analyses.

The genetic diversity analyses revealed heterogeneous patterns among populations. The rapid LD decay observed in wild yak and Indian yak suggests relatively high genomic diversity and/or weaker effects of recent artificial selection (37, 38). This pattern is expected for wild yak, which has experienced less intensive domestication and breeding management than domestic populations. In contrast, Leiwuqi yak showed relatively high nucleotide diversity, indicating that this population still retains abundant genetic variation. This may be associated with its long-term adaptation to the complex ecological conditions of eastern Tibet and possible historical gene flow with other yak populations.

ROH-based analyses provided additional information on demographic history and inbreeding (39). The presence of short ROH fragments in Leiwuqi yak and Datong yak may reflect ancient inbreeding or historical founder effects, whereas longer ROH fragments are generally indicative of more recent inbreeding (40, 41). The relatively high inbreeding coefficient observed in Leiwuqi yak suggests that, despite its high nucleotide diversity, the population may have experienced recent local isolation, limited breeding exchange, or small effective population size. These findings highlight the need to maintain genetic variation and avoid further inbreeding in future conservation and breeding programs.

Population structure analyses showed that Chinese domestic yak populations, including Leiwuqi, Tianzhu, Hongyuan, and Datong yaks, largely overlapped with wild yak in the PCA, with no clear independent clustering among native Qinghai–Tibet Plateau yak populations. This pattern suggests a highly shared genetic background, which may reflect historical gene flow between wild and domestic yaks, as also reported in previous genomic studies (8, 42). Datong yak is an artificially bred cultivar developed by crossing domestic yak with wild yak, and it possesses excellent production performance (5). Long-term sympatric distribution, traditional grazing systems, and occasional natural hybridization may have further promoted genetic exchange among local yak populations (12). In contrast, Indian yak formed a distinct cluster and showed clear genetic differentiation from Chinese yak germplasm resources. This differentiation may be associated with geographic isolation, ecological differences, and independent demographic or breeding histories. The ADMIXTURE results were consistent with the PCA, showing a genetic boundary between Indian yak and Chinese yak populations (18), whereas Chinese indigenous yak populations exhibited relatively homogeneous ancestral components. These results suggest that geographic separation, rather than breed differentiation among Chinese local yak populations, represents the major axis of genetic differentiation in this dataset.

Although Leiwuqi yak did not form a completely independent genetic cluster in PCA and ADMIXTURE analyses, this does not exclude the presence of population-specific genomic features. Local adaptation can occur at specific genomic regions even when populations share a broadly similar genome-wide genetic background, as divergent selection may generate localized genomic differentiation despite gene flow or genome-wide similarity (43–45). Such genome-wide similarity may obscure fine-scale population differentiation, whereas natural selection can still shape adaptive genetic variation at functionally important loci. Therefore, the germplasm characteristics of Leiwuqi yak should be further elucidated from the perspectives of regional genomic differentiation, genetic diversity patterns, and candidate loci under selection.

Our results revealed that Leiwuqi yak and Datong yak shared genomic regions with high nucleotide diversity, which were significantly enriched in pathways related to olfactory perception and chemical stimulus recognition. Olfactory receptor genes constitute one of the largest and most rapidly evolving gene families in mammals (46, 47), and are widely involved in essential biological processes such as environmental perception, food identification, social communication, and reproductive behavior (48, 49). For herbivorous livestock inhabiting alpine and cold environments, accurate recognition of plant food resources, water sources, predators, and social or reproduction-related signals is particularly important. Therefore, the enrichment of olfaction-related genes in high-diversity regions suggests that genetic variation associated with environmental perception has been effectively retained in yak populations. In addition to genes associated with environmental perception, several shared candidate genes with distinct biological functions were also identified. MMP1 is involved in extracellular matrix remodeling and tissue repair (50), while LOXL2 participates in collagen cross-linking, connective tissue development, hypoxia adaptation, and fibrosis regulation (51–53). PGR plays a central role in reproductive regulation (54). These genes suggest that the maintenance of genetic variation in yak populations may involve multiple biological pathways, including environmental perception, tissue remodeling, hypoxia response, and reproductive regulation.

To further explore the unique population-specific adaptive evolutionary characteristics of Leiwuqi yak, we performed a selective sweep analysis based on the nucleotide diversity ratio and population differentiation coefficient between Leiwuqi yak and other domestic yak populations. A total of 466 coding genes were identified within the candidate selected regions, indicating the presence of localized genomic differentiation in Leiwuqi yak. Functional enrichment analysis showed that these genes were mainly enriched in the Wnt signaling pathway, the NF-kappa B signaling pathway, and cancer-related pathways. Among them, the Wnt signaling pathway is involved in organismal development, tissue homeostasis, and reproductive regulation (55, 56), while the NF-κB signaling pathway plays important roles in immune responses and stress responses (57). GO terms related to light absorption and receptor-mediated endocytosis further indicate that visual perception, environmental sensing, and cellular response mechanisms may participate in the adaptive differentiation of Leiwuqi yak, as supported by previous genomic studies of yak adaptation to the high-altitude environment (58).

It should be acknowledged that the newly sequenced LWY samples (mean depth 3.87×) and the publicly available genomes (mean depth 9.04×) differed in sequencing depth, which may potentially affect genotype quality and downstream analyses. To minimize this bias, all samples were processed through a unified pipeline with stringent filtering criteria. Moreover, the consistency of results across multiple independent methods supports the robustness of our main conclusions.

## Conclusion

This study characterized the genetic diversity, population structure, and candidate genomic regions potentially associated with local adaptation of Leiwuqi yak using whole-genome resequencing. Leiwuqi yak retained relatively abundant genetic diversity but showed evidence of potential inbreeding. Population structure analyses revealed a broadly shared genetic background among Chinese domestic yak populations, while Indian yak showed clear differentiation. Selective sweep analysis identified 466 candidate genes potentially associated with local adaptation in Leiwuqi yak, mainly involving development, immune response, environmental sensing, and cellular regulation. These findings provide valuable genomic information for the conservation, evaluation, and sustainable utilization of Leiwuqi yak.

## Data Availability

Whole-genome resequencing data of samples from 110 Leiwuqi yaks were submitted to the NCBI database (https://www.ncbi.nlm.nih.gov/) in this study, and the corresponding project has been registered in NCBI BioProject under accession number PRJNA1472617. All accession numbers of the samples are listed in Supplementary Table S2.

## Footnotes

1
https://www.omicshare.com/tools/

## References

[1] Xiangdong ZI Bin L Wei X Yucai Z Xianrong X Jian LI . Transcriptomic analysis of IVF embryonic development in the yak (*Bos grunniens*) via RNA-seq. Sci Agric Sin. (2018) 51:1577–89. doi: 10.3864/j.issn.0578-1752.2018.08.015

[2] Mamet T Xu B Li X Zhang J Li C Wang L. Chemical and nutritional composition of Pamir yak milk from Xinjiang. J Anim Physiol Anim Nutr (Berl). (2023) 107:350–6. doi: 10.1111/jpn.13717, 35522695

[3] Li K Shahzad M Zhang H Jiang X Mehmood K Zhao X . Socio-economic burden of parasitic infections in yaks from 1984 to 2017 on Qinghai Tibetan plateau of China–a review. Acta Trop. (2018) 183:103–9. doi: 10.1016/j.actatropica.2018.04.011, 29626434

[4] Mahar K Gurao A Kumar A Singh LP Chitkara M Gowane GR . Genomic inbreeding analysis reveals resilience and genetic diversity in Indian yak populations. Gene. (2024) 928:148787. doi: 10.1016/j.gene.2024.148787, 39053660

[5] Liu Y Luo J Dou J Yan B Ren Q Tang B . The sequence and *de novo* assembly of the wild yak genome. Sci Data. (2020) 7:66. doi: 10.1038/s41597-020-0400-3, 32094352 PMC7039982

[6] Wang H Zhong J Zhang C Chai Z Cao H Wang J . The whole-transcriptome landscape of muscle and adipose tissues reveals the cerna regulation network related to intramuscular fat deposition in yak. BMC Genomics. (2020) 21:1–15. doi: 10.1186/s12864-020-6757-z, 32381004 PMC7203869

[7] Jiang H Chai Z Cao H Zhang C Zhu Y Zhang Q . Genome-wide identification of snps associated with body weight in yak. BMC Genomics. (2022) 23:1–12. doi: 10.1186/s12864-022-09077-4, 36522700 PMC9756674

[8] Qiu Q Wang L Wang K Yang Y Ma T Wang Z . Yak whole-genome resequencing reveals domestication signatures and prehistoric population expansions. Nat Commun. (2015) 6:1–7. doi: 10.1038/ncomms10283, 26691338 PMC4703879

[9] Jia J Liang C Wu X Xiong L Bao P Chen Q . Effect of high proportion concentrate dietary on yak jejunal structure, physiological function and protein composition during cold season. Sci Rep. (2021) 11:1–9. doi: 10.1038/s41598-021-84991-3, 33750879 PMC7970894

[10] Qiu Q Zhang G Ma T Qian W Wang J Ye Z . The yak genome and adaptation to life at high altitude. Nat Genet. (2012) 44:946–9. doi: 10.1038/ng.2343 22751099

[11] Zhang Q Wang Q Zhang Y Cheng S Hu J Ma Y . Comprehensive analysis of microRNA-messenger RNA from white yak testis reveals the differentially expressed molecules involved in development and reproduction. Int J Mol Sci. (2018) 19:3083. doi: 10.3390/ijms19103083, 30304826 PMC6213350

[12] Sivalingam J Vineeth MR Surya T Singh K Dixit SP Niranjan SK . Genomic divergence reveals unique populations among Indian yaks. Sci Rep. (2020) 10:3636. doi: 10.1038/s41598-020-59887-3, 32108137 PMC7046631

[13] Kang Y Guo S Wang X Cao M Pei J Li R . Whole-genome resequencing highlights the unique characteristics of Kecai yaks. Animals (Basel). (2022) 12:2682. doi: 10.3390/ani12192682, 36230423 PMC9559661

[14] Li G Luo J Wang F Xu D Ahmed Z Chen S . Whole-genome resequencing reveals genetic diversity, differentiation, and selection signatures of yak breeds/populations in Qinghai, China. Front Genet. (2022) 13:1034094. doi: 10.3389/fgene.2022.1034094, 36704337 PMC9871260

[15] Cai B Wu X Shi Y Kang Y Ding Z Guo S . Whole-genome sequencing unveils the uniqueness of Yushu yaks (*bos grunniens*). Int J Mol Sci. (2025) 26:3879. doi: 10.3390/ijms26083879, 40332613 PMC12027638

[16] Ji Q Xin J Chai Z Zhang C Dawa Y Luo S . A chromosome-scale reference genome and genome-wide genetic variations elucidate adaptation in yak. Mol Ecol Resour. (2021) 21:201–11. doi: 10.1111/1755-0998.13236, 32745324 PMC7754329

[17] Zhang S Li J Zhao Y Tang Y Li H Song T . Whole-genome resequencing reveals genetic diversity, differentiation, and selection signatures of yak breeds/populations in Southwestern China. Front Genet. (2024) 15:1382128. doi: 10.3389/fgene.2024.1382128 38873117 PMC11169580

[18] Ahmad SF Gangwar M Kumar A Kumar A Dige MS Jha GK . Dissecting genomes of multiple yak populations: unveiling ancestry and high-altitude adaptation through whole-genome resequencing analysis. BMC Genomics. (2025) 26:214. doi: 10.1186/s12864-025-11387-2, 40033180 PMC11877770

[19] Medugorac I Graf A Grohs C Rothammer S Zagdsuren Y Gladyr E . Whole-genome analysis of introgressive hybridization and characterization of the bovine legacy of Mongolian yaks. Nat Genet. (2017) 49:470–5. doi: 10.1038/ng.3775 28135247

[20] Chen S Zhou Y Chen Y Gu J. Fastp: an ultra-fast all-in-one FASTQ preprocessor. Bioinformatics. (2018) 34:i884–90. doi: 10.1093/bioinformatics/bty560, 30423086 PMC6129281

[21] Li H Durbin R. Fast and accurate short read alignment with burrows-wheeler transform. Bioinformatics. (2009) 25:1754–60. doi: 10.1093/bioinformatics/btp324, 19451168 PMC2705234

[22] Li H Handsaker B Wysoker A Fennell T Ruan J Homer N . The sequence alignment/map format and samtools. Bioinformatics. (2009) 25:2078–9. doi: 10.1093/bioinformatics/btp352, 19505943 PMC2723002

[23] Muema DM Mthembu M Schiff AE Singh U Corleis B Chen D . Contrasting inflammatory signatures in peripheral blood and bronchoalveolar cells reveal compartment-specific effects of HIV infection. Front Immunol. (2020) 11:864. doi: 10.3389/fimmu.2020.00864, 32508817 PMC7248324

[24] Garcia-Alcalde F Okonechnikov K Carbonell J Cruz LM Goetz S Tarazona S . Qualimap: evaluating next-generation sequencing alignment data. Bioinformatics. (2012) 28:2678–9. doi: 10.1093/bioinformatics/bts503 22914218

[25] Mckenna A Hanna M Banks E Sivachenko A Cibulskis K Kernytsky A . The genome analysis toolkit: a mapreduce framework for analyzing next-generation DNA sequencing data. Genome Res. (2010) 20:1297–303. doi: 10.1101/gr.107524.110, 20644199 PMC2928508

[26] Danecek P Auton A Abecasis G Albers CA Banks E Depristo MA . The variant call format and vcftools. Bioinformatics. (2011) 27:2156–8. doi: 10.1093/bioinformatics/btr330, 21653522 PMC3137218

[27] Zhang C Dong S Xu J He W Yang T. Poplddecay: a fast and effective tool for linkage disequilibrium decay analysis based on variant call format files. Bioinformatics. (2019) 35:1786–8. doi: 10.1093/bioinformatics/bty875, 30321304

[28] Patterson N Price AL Reich D. Population structure and eigenanalysis. PLoS Genet. (2006) 2:e190–3. doi: 10.1371/journal.pgen.0020190, 17194218 PMC1713260

[29] Alexander DH Lange K. Enhancements to the admixture algorithm for individual ancestry estimation. BMC Bioinform. (2011) 12:1–6. doi: 10.1186/1471-2105-12-246, 21682921 PMC3146885

[30] Hu R Zou H Wang Z Cao B Peng Q Jing X . Nutritional interventions improved rumen functions and promoted compensatory growth of growth-retarded yaks as revealed by integrated transcripts and microbiome analyses. Front Microbiol. (2019) 10:318. doi: 10.3389/fmicb.2019.00318, 30846981 PMC6393393

[31] Zhu Y Li X Lousang-Zhaxi Suolang-Zhaxi Suolang Ciyang . House feeding pattern increased male yak fertility by improving gut microbiota and serum metabolites. Front Vet Sci. (2022) 9:989908. doi: 10.3389/fvets.2022.989908 36118356 PMC9478890

[32] Kour A Niranjan SK Malayaperumal M Surati U Pukhrambam M Sivalingam J . Genomic diversity profiling and breed-specific evolutionary signatures of selection in Arunachali yak. Genes (Basel). (2022) 13:254. doi: 10.3390/genes13020254, 35205299 PMC8872319

[33] Cooper GM Stone EA Asimenos G Green ED Batzoglou S Sidow A. Distribution and intensity of constraint in mammalian genomic sequence. Genome Res. (2005) 15:901–13. doi: 10.1101/gr.3577405, 15965027 PMC1172034

[34] Kryukov GV Pennacchio LA Sunyaev SR. Most rare missense alleles are deleterious in humans: implications for complex disease and association studies. Am J Hum Genet. (2007) 80:727–39. doi: 10.1086/513473, 17357078 PMC1852724

[35] Depristo MA Banks E Poplin R Garimella KV Daly MJ. A framework for variation discovery and genotyping using next-generation DNA sequencing data. Nat Genet. (2011) 43:491–8. doi: 10.1038/ng.806 21478889 PMC3083463

[36] Van der Auwera GA Carneiro MO Hartl C Poplin R Del Angel G Levy‐Moonshine A . From FastQ data to high-confidence variant calls: the genome analysis toolkit best practices pipeline. Curr Protoc Bioinformatics. (2013) 43:11.10.1–11.10.33. doi: 10.1002/0471250953.bi1110s43 PMC4243306 25431634

[37] Shi H Li T Su M Wang H Li Q Lang X . Whole genome sequencing revealed genetic diversity, population structure, and selective signature of panou tibetan sheep. BMC Genomics. (2023) 24:50. doi: 10.1186/s12864-023-09146-2, 36707771 PMC9883975

[38] Ali N Li D Eltahawy MS Abdulmajid D Hong D. Mining of favorable alleles for seed reserve utilization efficiency in *Oryza sativa* by means of association mapping. BMC Genet. (2020) 21:4. doi: 10.1186/s12863-020-0811-3, 31948408 PMC6966888

[39] Kuec ID Bokovi I Zorc M Gvozdanovi K Kuec G. Genomic characterization of the Istrian shorthaired hound. Animals (Basel). (2020) 10:2013. doi: 10.3390/ani10112013 33139624 PMC7693797

[40] Kirin M Mcquillan R Franklin CS Campbell H Mckeigue PM Wilson JF. Genomic runs of homozygosity record population history and consanguinity. PLoS One. (2010) 5:e13996. doi: 10.1371/journal.pone.0013996, 21085596 PMC2981575

[41] Marras G Gaspa G Sorbolini S Dimauro C Ajmone-Marsan P Valentini A . Analysis of runs of homozygosity and their relationship with inbreeding in five cattle breeds farmed in Italy. Anim Genet. (2015) 46:110–21. doi: 10.1111/age.12259, 25530322

[42] Habtamu G Xiaoyun W Min C Pengjia B Xuezhi D Ping Y. Copy number variations of *Klf6* modulate gene transcription and growth traits in Chinese Datong yak (*Bos grunniens*). Animals (Basel). (2018) 8:145. doi: 10.3390/ani8090145 30134528 PMC6162419

[43] Nosil P Funk DJ Arrientos DO. Divergent selection and heterogeneous genomic divergence. Mol Ecol. (2010) 18:375–402. doi: 10.1111/j.1365-294X.2008.03946.x 19143936

[44] Savolainen O Lascoux M Meril J. Ecological genomics of local adaptation. Nat Rev Genet. (2013) 14:807–20. doi: 10.1038/nrg3522 24136507

[45] Via S. Divergence hitchhiking and the spread of genomic isolation during ecological speciation-with-gene-flow. Philos Trans R Soc Lond. (2012) 367:451–60. doi: 10.1098/rstb.2011.0260, 22201174 PMC3233719

[46] Yoshihito N Atsushi M Kazushige T. Acceleration of olfactory receptor gene loss in primate evolution: possible link to anatomical change in sensory systems and dietary transition. Mol Biol Evol. (2018) 35:1437–50. doi: 10.1093/molbev/msy042 29659972

[47] Lee K Nguyen DT Choi M Cha SY Kim JH Dadi H . Analysis of cattle olfactory subgenome: the first detail study on the characteristics of the complete olfactory receptor repertoire of a ruminant. BMC Genomics. (2013) 14:596. doi: 10.1186/1471-2164-14-596, 24004971 PMC3766653

[48] Khoury S Gudziol V Grégoire S Cabaret S Menzel S Martine L . Lipidomic profile of human nasal mucosa and associations with circulating fatty acids and olfactory deficiency. Sci Rep. (2021) 11:16771. doi: 10.1038/s41598-021-93817-1, 34408170 PMC8373950

[49] Doyle WI Meeks JP. Excreted steroids in vertebrate social communication. J Neurosci. (2018) 38:3377–87. doi: 10.1523/JNEUROSCI.2488-17.2018 29519850 PMC5895034

[50] Bhattacharya A Janal MN Veeramachaneni R Dolgalev I Albertson DG. Oncogenes overexpressed in metastatic oral cancers from patients with pain: potential pain mediators released in exosomes. Sci Rep. (2020) 10:14724. doi: 10.1038/s41598-020-71298-y, 32895418 PMC7477576

[51] Yang J Savvatis K Kang JS Fan P Zhong H Schwartz K . Targeting LOXL2 for cardiac interstitial fibrosis and heart failure treatment. Nat Commun. (2016) 7:13710. doi: 10.1038/ncomms13710, 27966531 PMC5171850

[52] Zhao Y Tang K Tianbao X Wang J Yang J Li D. Increased serum lysyl oxidase-like 2 levels correlate with the degree of left atrial fibrosis in patients with atrial fibrillation. Biosci Rep. (2017) 37:BSR20171332. doi: 10.1042/BSR20171332, 29089463 PMC5696452

[53] Wang M Zhao X Zhu D. Hif-1α promoted vasculogenic mimicry formation in hepatocellular carcinoma through LOXL2 up-regulation in hypoxic tumor microenvironment. J Exp Clin Cancer Res. (2017) 36:60. doi: 10.1186/s13046-017-0533-1, 28449718 PMC5408450

[54] Demayo FJ Lydon JP. 90 years of progesterone: new insights into progesterone receptor signaling in the endometrium required for embryo implantation. J Mol Endocrinol. (2020) 65:T1–T14. doi: 10.1530/jme-19-0212, 31809260 PMC7261627

[55] Yang Y Yu-Chen Y Yan C Jun-Long Z Chun-Chen G Hua H . Crosstalk between hepatic tumor cells and macrophages via Wnt/β-catenin signaling promotes M2-like macrophage polarization and reinforces tumor malignant behaviors. Cell Death Dis. (2018) 9:793. doi: 10.1038/s41419-018-0818-0 30022048 PMC6052107

[56] Hirakawa T Nasu K Miyabe S Kouji H Narahara H. Β-catenin signaling inhibitors ICG-001 and C-82 improve fibrosis in preclinical models of endometriosis. Sci Rep. (2019) 9:20056. doi: 10.1038/s41598-019-56302-4, 31882904 PMC6934788

[57] Zhang X Chen L Zheng F Du Y. The efficacy of microsurgery in the treatment of cerebral aneurysm rupture and its effect on nf-κb, mcp-1 and mmp-9. Exp Ther Med. (2017) 14:3744–8. doi: 10.3892/etm.2017.4928, 29042973 PMC5639380

[58] Yang X Cui Y Yue J He H Meng Y. The histological characteristics, age-related thickness change of skin, and expression of the HSPs in the skin during hair cycle in yak (*Bos grunniens*). PLoS One. (2017) 12:e0176451. doi: 10.1371/journal.pone.0176451, 28463974 PMC5413005

